# Pregnancy Outcomes Following Planned Cesarean Section: Experience From a Tertiary Care Hospital in Bahrain

**DOI:** 10.7759/cureus.86994

**Published:** 2025-06-29

**Authors:** Laxmi Saha, Maha Ghorabah, Reham Fathi Hozayan, Nusaiba Ibrahim, Yasmin Abozenah

**Affiliations:** 1 Obstetrics and Gynaecology, George Eliot Hospital, Nuneaton, GBR; 2 Obstetrics and Gynaecology, King Hamad University Hospital, Busaiteen, BHR; 3 Obstetrics and Gynaecology/Reproductive Science, Yale University, New Haven, USA

**Keywords:** elective repeat cesarean section(ercs), emergency and elective cesarean, gestational age, neonatal outcomes, spontaneous labor

## Abstract

Introduction: While current clinical guidelines recommend scheduling repeat cesarean sections (CSs) at or after 39 weeks of gestation to optimize maternal and neonatal outcomes, emergent circumstances, such as spontaneous labor or acute maternal or fetal concerns, may necessitate performing the procedure prior to reaching this gestational age. This study aimed to compare maternal and neonatal outcomes associated with repeat CSs performed either before or at/after 39 weeks of gestation. In addition, it identified the indications of emergency repeat cesarean sections (EmRCS). The findings are expected to inform clinical decision-making and contribute to improved maternal and neonatal outcomes.

Methods: A retrospective review was conducted on repeat CSs at King Hamad University Hospital, Bahrain, from 2017 to 2021. Deliveries were categorized by gestational age (<39 weeks vs. ≥39 weeks) and by type, elective repeat cesarean section (ERCS) versus EmRCS. Statistical analyses, including chi-square tests, t-tests, and logistic regression, were used to assess associations between spontaneous labor, maternal characteristics, and neonatal outcomes.

Results: Among 1421 women, 330 (23%) underwent repeat CSs at or beyond 39 weeks, while 235 (17%) were classified as EmRCS. A significantly higher incidence of EmRCS was observed in deliveries at or after 39 weeks (P < 0.05). Notably, spontaneous labor was responsible for 55% (n = 137) of EmRCS cases and was weakly negatively correlated with body mass index (r = -0.07, p = 0.012) and positively correlated with the number of previous CSs (r = 0.116, p < 0.001). Logistic regression indicated that women with more than one previous CS had increased odds of entering spontaneous labor (OR 2.36, 95% CI 1.41-3.81; p = 0.001). Moreover, 9% of neonates (n = 128) required neonatal intensive care unit (NICU) admission, with a significantly higher rate among those delivered before 39 weeks (p = 0.001). No significant differences were observed between elective CSs performed before or after 39 weeks.

Conclusion: ERCS scheduled at 39 weeks appears to reduce the risk of neonatal respiratory complications. However, the risk of spontaneous labor, leading to emergency repeat procedures, remains a concern, particularly among women with multiple previous CSs. These findings underscore the importance of individualized patient counseling and delivery planning to balance the benefits of fetal maturity against the risks associated with early, unplanned delivery.

## Introduction

The rising incidence of primary cesarean sections (CSs) has led to an increase in repeat cesarean deliveries. Elective repeat cesarean section (ERCS) is typically scheduled at 39 weeks of gestation to minimize neonatal complications such as respiratory morbidities and the subsequent need for admission to a neonatal Intensive care unit (NICU) [1,2]. However, spontaneous labor, which is inherently unpredictable, may occur before reaching this gestational milestone [3]. When spontaneous labor ensues before a planned repeat CS, the procedure may need to be converted to an emergency repeat cesarean section (EmRCS), a scenario that research indicates is accompanied by a higher incidence of complications for both the mother and the newborn [4,5]. Furthermore, EmRCS is associated with a more profound psychological impact on the mother, potentially increasing the risk of post-traumatic stress disorder [6-9]. It is also important to note that repeat CSs are generally more complex than primary ones, carrying greater intraoperative risks such as extended operative times and increased chances of injury to adjacent viscera like the bladder and ureters [10-13]. These challenges are compounded during off-peak hours, for instance, after midnight, when consultant availability may be delayed and existing medical staff are at risk of fatigue due to high workloads in busy delivery suites.

Understanding the outcomes of CSs performed before and after 39 weeks of gestation, along with identifying factors that predispose to spontaneous labor prior to this timeframe, is crucial for determining the optimal timing for ERCS. Moreover, these findings provide essential information for pregnant women, enabling them to understand the gestational age-related risks and engage in effective shared decision-making regarding their care. Despite the importance of this issue, the current evidence in this area remains relatively scarce. Therefore, this study was designed to compare the outcomes of cesarean deliveries conducted before 39 weeks with those performed at or after 39 weeks of gestation and to identify risk factors that heighten the likelihood of spontaneous labor onset.

## Materials and methods

Study design and population 

This retrospective, comparative study was conducted at King Hamad University Hospital, Bahrain. Hospital records from 2017 to 2021 were reviewed to identify all repeat CSs performed between 37 and 41 weeks of gestation. Cases were stratified into two groups based on gestational age at the time of CSs: those conducted before 39 weeks and those performed at or after 39 weeks. Participants were matched according to relevant parameters, including age, medical history, and gestational age, to minimize confounding. Data extracted included demographic details, details of the CSs type (elective or emergency), indications for EmRCS, and maternal and neonatal outcomes. Table 1 describes the inclusion and exclusion criteria for the study population.

**Table 1:** Inclusion and exclusion criteria of the study population APH: antepartum hemorrhage, CS: cesarean section, EmRCS: emergency repeat cesarean section, FGR: fetal growth restriction, PET: preeclampsia, SCT: sacrococcygeal teratoma, UA: umbilical artery

Population criteria	Inclusion	Exclusion
Study population	All repeat CS booked for CS	Repeat CS not booked for CS and underwent EmRCS
Number of fetuses	Singleton pregnancy	Multiple pregnancy
Gestations	Gestation from 37 weeks and onward	Gestation before 37 weeks
Maternal comorbidities	No serious maternal comorbidities	Serious maternal comorbidities, like major APH/placenta previa, severe PET/eclampsia, significant cardiac disease
Fetal condition	No major fetal structural abnormalities or fetal compromise was evident in the UA Doppler scan.	Major structural abnormalities that can affect fetal outcomes like SCT, encephalopathy, or severe FGR/fetal compromise are evident in UA Doppler.

Study outcomes 

The primary outcome was the gestational age at which the repeat CS was performed. Specifically, the study compared the frequency of ERCS versus EmRCS, as well as the associated maternal and fetal outcomes across the two gestational groups.

Secondary outcomes included comparative maternal and neonatal outcomes following ERCS and EmRCS, indications leading to EmRCS, and identification of risk factors associated with the spontaneous onset of labor prior to scheduled ERCS.

Statistical analysis 

Descriptive statistics (frequency analysis) were utilized to summarize the primary and secondary outcomes. A multivariate regression model was constructed to evaluate the risk factors associated with spontaneous labor, which was the primary indication for EmRCS. All statistical tests were two-tailed with a significance level set at p < 0.05.

## Results

Frequency and outcome of repeat CS performed before and after 39 weeks of gestation

A total of 1,421 cases were eligible for the study. Of these, 313 (23%) repeat CSs were performed at or after 39 weeks of gestation. Table 2 compares the results of repeat CS performed before and after 39 weeks of gestation. Women in the <39-week group were significantly older (32.62 ± 5.04 years vs. 30.84 ± 5.14 years, p < 0.05). No significant difference was observed in body mass index (BMI) between groups (31.62 ± 10.80 vs. 31.22 ± 6.10, p = 0.885), with over half of the participants classified as obese. Although maternal comorbidities and the type of previous CS did not differ significantly between the groups, a considerably higher proportion of women with two or more previous CS were in the <39-week group (51.9% vs. 30.6%, p < 0.05). Additionally, CSs performed before 39 weeks were more frequently conducted during out-of-office hours (10.7% vs. 6.7%, p = 0.034). Maternal complications were uncommon (overall rate of 1.75%) and did not differ significantly between groups. In contrast, fetal outcomes varied significantly: neonates delivered before 39 weeks required higher rates of NICU admission (10.4% vs. 4.5%, p = 0.001), although NICU admission rates after elective repeat CS were comparable across groups (6.7% vs. 6.5%, p = 0.944).

**Table 2:** Outcomes of repeat CS performed before and after 39 weeks of gestation *Statistically significant BMI: body mass index, CS: cesarean section, NICU: neonatal intensive unit

Variables	CS done before 39 weeks	CS done after 39 weeks or more	P-value
Age	32.62 ± 5.04	30.84 ± 5.14	0.000*
BMI	31.62 ± 10.80	31.22 ± 6.10	0.885
Maternal comorbidities			0.226
Yes	465 (42.6%)	128 (38.8%)	
No	626 (57.4%)	202 (61.2%)	
Previous CS			0.000*
1	525 (48.1%)	229 (69.4%)	
≥2	566 (51.9%)	101 (30.6%)	
Type of previous CS			0.142
Elective	517 (47.4%)	147 (44.5%)	
Emergency	391 (35.8%)	137 (41.5%)	
Unknown	183 (16.8%)	46 (15.0%)	
Type of current CS			0.004*
Elective	884 (81.0%)	290 (87.9%)	
Emergency	207 (19.0%)	40 (12.1%)	
Time of surgeries			0.034*
Office hour	974 (89.3%)	308 (93.3%)	
Out of office	117 (10.7%)	22 (6.7%)	
Maternal outcome			0.809
Uneventful	1073 (98.4%)	324 (98.2%)	
Eventful	18 (1.6%)	6 (1.8%)	
Fetal outcome			0.001*
No admission	978 (89.6%)	315 (95.5%)	
NICU admission	113 (10.4%)	15 (4.5%)	
NICU admission after elective CS	59 (6.7%)	19 (6.5%)	0.944

Outcomes of ERCS and EmRCS

Of the total cases, 247 (17.38%) CSs were performed as emergency procedures. A comparison of the outcome of repeat CS for elective and emergency cases is provided in Table 3. A statistically significant increase in emergency CSs was noted during out-of-office hours (p < 0.05). Additionally, 667 women (47% of the study population) had a history of more than one previous CS. Notably, emergency procedures were significantly more common among those with two or more previous CSs (p < 0.05). The difference in maternal outcomes between elective and emergency cesarean deliveries approached statistical significance (p = 0.053), while neonates delivered by emergency CS had a significantly higher rate of NICU admissions (p < 0.05).

**Table 3:** Outcomes of ERCS and EmRCS *Statistically significant CS: cesarean section, NICU: neonatal intensive unit, EmRCS: emergency repeat cesarean sections, ERCS: elective repeat cesarean section

Variables	Elective (n=1174)	Emergency (n=247)	P-value
Time of surgeries			0.000*
Office hours	1130 (96.3%)	152 (61.5%)	
Out of office hours	44 (3.7%)	95 (38.5%)	
Previous CS			0.001*
1	647 (55.1%)	107 (43.3%)	
≥2	527 (44.9%)	140 (56.7%)	
Maternal outcome			0.053
Uneventful	1158 (98.6%)	239 (96.8%)	
Eventful	16 (1.4%)	8 (3.2%)	
Fetal outcome			0.000*
No admission	1096 (93.4%)	197 (79.8%)	
NICU admission	78 (6.6%)	50 (20.2%)	

**Figure 1:**
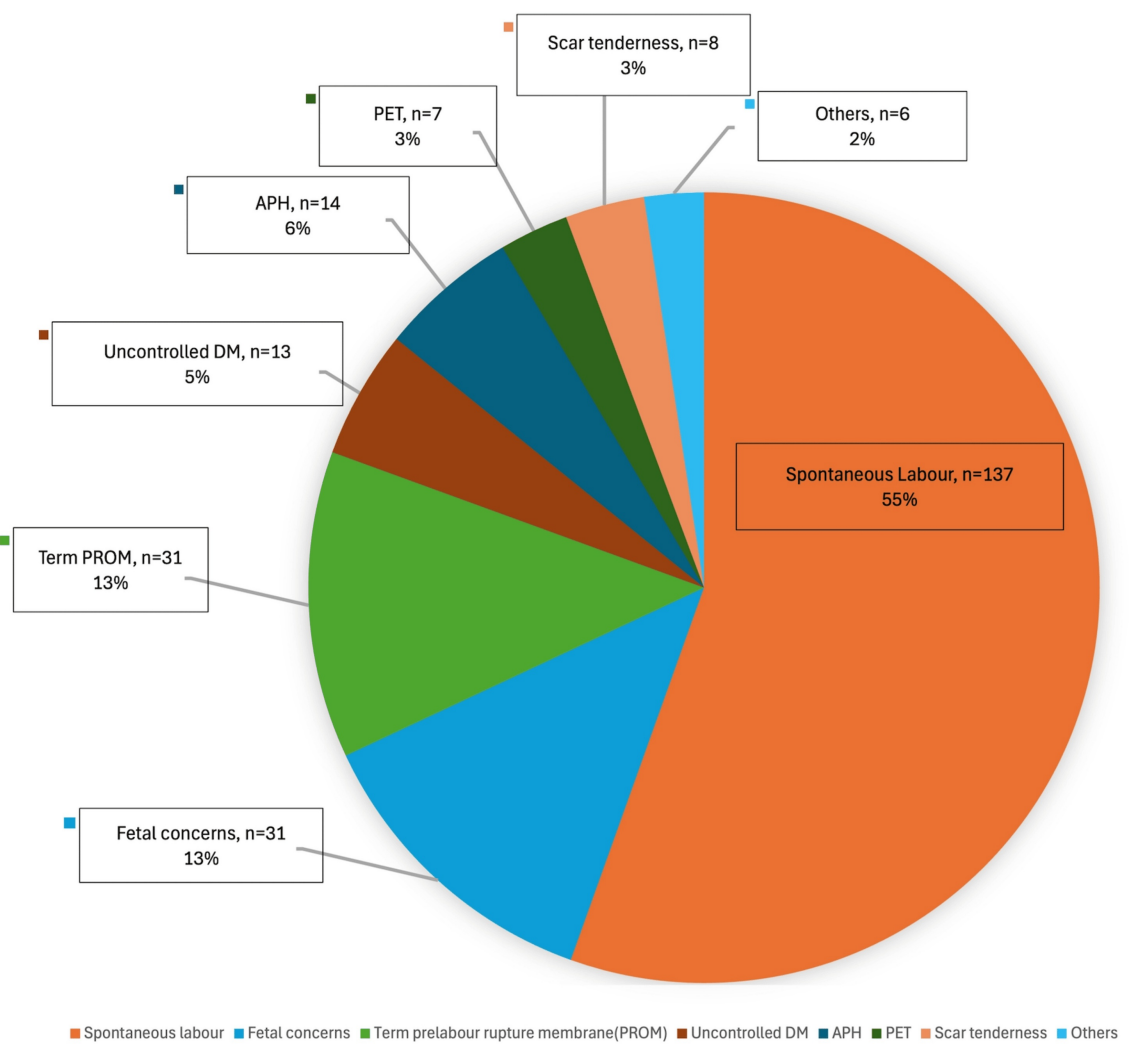
Indications of EmRCS APH: antepartum hemorrhage, EmRCS: emergency repeat cesarean section, PET: preeclampsia, DM: diabetes mellitus

Figure 1 shows that half of the EmRCS (n = 137, 55%) were performed due to spontaneous labor. Other significant indications for EmRCS included fetal concerns (n = 31), term pre-labor rupture of membranes (PROM) (n = 31), antepartum hemorrhage (APH) (n = 14), and uncontrolled diabetes mellitus (n = 13). 

Table 4 shows the correlation analysis between spontaneous labor and five maternal factors. This bivariate analysis revealed a modest but statistically significant negative correlation between BMI and spontaneous labor (r = -0.07, p = 0.012) and a positive correlation between the number of previous CSs and spontaneous labor (r = 0.116, p = 0.00). Furthermore, the regression model indicated that women with two or more previous CSs had significantly higher odds of experiencing spontaneous labor (OR 2.36, 95% CI 1.41-3.81, p = 0.001).

**Table 4:** Bivariate analysis of spontaneous labor and different maternal factors *Statistically significant BMI: body mass index, Sig: significant

		Age	BMI	Previous CS (emergency)	Maternal comorbidity	Previous CS ≥2
Spontaneous labor	Correlation coefficient	0.049	-0.078	0.019	-0.006	0.116
	Sig. (2-tailed)	0.065	0.012*	0.521	0.831	0.000*

## Discussion

The timing of repeat cesarean section in real-life situations

Globally, the incidence of CSs is rising, and repeat CS is increasingly common in obstetric practice. In our study, approximately two-thirds of women underwent ERCS before 39 weeks, reflecting a deviation from guidelines put forth by professional bodies [1,2,14]. This deviation was associated with a higher rate of NICU admissions for infants delivered before 39 weeks compared to those delivered at or after 39 weeks. These findings align with prior studies reporting a high incidence of CSs performed before 39 weeks [3-5].

Factors contributing to spontaneous labor before 39 weeks

Spontaneous labor emerged as the primary indication for EmRCS in our cohort. In addition, factors such as premature rupture of membranes, fetal distress, antepartum hemorrhage, and discomfort at previous cesarean incision sites contributed to the decision for EmRCS (Figure 1). Literature indicates that approximately 10% of women may experience spontaneous labor before 39 weeks [3], and this likelihood increases in the presence of risk factors such as a history of preterm birth or preterm rupture of membranes and short interpregnancy intervals [15]. Although our analysis did not investigate all potential contributing factors, Table 4 shows that low BMI and a higher number of previous cesarean sections are significant risk factors for spontaneous labor. However, the weak correlations (all coefficients near zero) caution us against overinterpreting the clinical impact of these associations in isolation. Further multivariate analysis could help clarify how these factors interact with other variables and influence labor outcomes overall. Clinically, the threshold to perform a repeat CS is relatively low in term pregnancies, especially when there is concern about scar dehiscence or fetal well-being, a strategy aimed at mitigating the risk of catastrophic uterine rupture [6]. In light of these risk factors, some clinicians advocate for scheduling repeat CS after 38 weeks rather than waiting until 39 weeks, an approach that may also reduce resource burdens during off-office hours [15].

Maternal and fetal outcomes of repeat CS before and after 39 weeks

Several studies have reported no statistically significant differences in maternal outcomes based on gestational age at delivery for full-term pregnancies [5,6]. However, Breslin et al. have observed higher rates of uterine atony, blood transfusions, endometritis, and wound infection with CS performed before 39 weeks compared to those at 39 weeks [4]. In our study, the overall incidence of maternal complications was low (1.7%), with most complications occurring in the CS performed before 39 weeks. Potential contributing factors include the increased likelihood of emergency procedures during off hours, associated maternal comorbidities, or anatomical factors such as a poorly formed lower uterine segment [4]. 

The neonatal outcomes, however, were markedly affected by the timing of the CS. The Royal College of Obstetricians and Gynaecologists (RCOG) has noted that neonatal respiratory morbidity is higher (approximately 6%) among infants born before 39 weeks [16]. Consistent with this observation, our study found significantly higher NICU admissions for babies delivered via emergency CS before 39 weeks relative to those delivered at or after 39 weeks. It is interesting to note that the NICU admission rates following elective cesarean deliveries did not differ significantly between the gestational groups, as shown in Table 2 and Table 3. These outcomes are comparable to other studies, which demonstrated no significant increase in NICU admissions for cesarean deliveries performed after 38 weeks [17,18].

Importance of individualized decision-making for the timing of ERCs

Although our findings support the recommendation for scheduling CS at 39 weeks, particularly given the lower NICU admission rates associated with EmRCS at this gestational age, they also underscore the complexity of the decision-making process. The type of CS (elective versus emergency) appears to have a significant impact on both maternal and neonatal outcomes, potentially more so than the gestational age alone in term infants. Hence, individualized decision-making that considers maternal history, fetal well-being, and the specific risks and benefits of different delivery timings is paramount.

This discussion integrates current guidelines, reinforces the clinical relevance of our findings, and underscores the need for personalized care strategies in managing repeat cesarean deliveries.

Limitations

Despite best efforts, our study has the following limitations. As a retrospective observational study, our analysis depended entirely on the accuracy and completeness of existing medical records. This design inherently limits the ability to establish causation. Certain variables that might influence the timing of spontaneous labor and outcomes were not accounted for. For example, gestational age at previous CSs, interpregnancy intervals, or other nuanced maternal risk factors were not included in the analysis. The exclusion of these factors limits our ability to fully control for confounding effects that might impact both maternal and neonatal outcomes. The types of neonatal morbidities, duration of NICU stay, and long-term neonatal outcomes were not assessed. Neonatal complications associated with pre-39-week deliveries may be underestimated due to this lack of granularity. The study’s findings are based on data from a single tertiary care hospital in Bahrain. This localized data may not be representative of practices and outcomes in different geographical areas or healthcare settings. Caution is warranted when generalizing these results to other populations. We classified spontaneous labor based on retrospective data, where practitioners may have applied different thresholds for clinical decision-making. Furthermore, this variability might lead to subjective interpretations, affecting correlation accuracy. Our study primarily focused on immediate maternal and neonatal outcomes. The lack of perspective on long-term maternal health or developmental outcomes in infants limits the scope of our conclusions regarding the overall impact of the timing of repeat CSs. Future research should address these limitations by including broader variables, stringent data collection procedures, and evaluating both short- and long-term outcomes.

## Conclusions

The majority of repeat CSs in this study were performed before 39 weeks of gestation. EmRCS were primarily indicated by spontaneous labor, a phenomenon that was more common in women with a lower BMI and a higher number of previous CSs. Neonates delivered by CS before 39 weeks exhibited significantly higher rates of NICU admissions compared to those delivered at or after 39 weeks. In contrast, elective CSs showed no significant difference in NICU admission rates between the two gestational age groups.

These findings highlight the important correlations between maternal characteristics, previous cesarean history, and the onset of spontaneous labor, offering valuable insights for clinical practice. Although overall maternal and neonatal morbidity rates were low, the increased rate of NICU admissions for infants delivered before 39 weeks underscores the potential risks associated with early delivery in repeat CSs. Further research is necessary to refine current guidelines and optimize the decision-making process regarding the timing of repeat cesarean deliveries. Ultimately, these results contribute to ongoing efforts to balance maternal and neonatal outcomes, with the goal of enhancing patient care and clinical outcomes in obstetric practice.
